# Fully automated beamline control system for XAS beamlines

**DOI:** 10.1107/S1600577518007518

**Published:** 2018-06-17

**Authors:** Stefan Mangold

**Affiliations:** a Institute for Photon Science and Synchrotron Radiation, Karlsruhe Institute of Technology, PO Box 3640, 76021 Karlsruhe, Germany

**Keywords:** XAS, control system, automation, beamline

## Abstract

Owing to the growing demand for complex *in situ* experiments, the handling of synchrotron experiments has become increasingly complicated. The control system at the XAS beamline of the Karlsruhe Institute of Technology synchrotron was automated based on a user-driven development cycle. By combining intelligent remote-controlled hardware and highly integrated software layers it is now possible to record data with a high level of reproducibility, eliminating human malperformance. The additional advantage of automation is the possibility of real 24/7 operation without tiring. This paper focuses on the options of optimization and enhancement in the data acquisition.

## Abstract 2

State-of-the-art beamline automation for XAS beamlines implemented at the synchrotron at Karlsruhe Institute of Technology is described.

## 1. Introduction   

More than 40 years after the introduction of X-ray absorption fine structure (XAFS) spectroscopy (Stumm von Bordwehr, 1989 ▸) the complexity of the user experiments but also the possibilities of beamlines have changed dramatically. Even rather simple beamlines for X-ray absorption spectroscopy (XAS) have nowadays up to 80 motors and use different sample environments depending on the demand of the users. There has been significant progress in the automation and the fabrication of components at beamlines and storage rings which leads to higher stability and reliability. The demand for complex *in situ* measurements has increased dramatically over the last two decades, and there is a strong need for measuring multiple absorption edges in direct sequence with continuous and/or step scans. Examples are measurements of complex catalyst and battery systems, where scientists wish to monitor changes in the local environment of several elements during reaction or charging. Different scan lengths and acquisition times are also essential requirements. Multiple sample positions and angles can avoid beam damage or help with self-absorption issues. The parameters of all detectors during scans should be adjustable or even automatically optimized to ensure best possible data quality. Automation can help the user by offering effective alignment procedures and a simple way to create a sequence of scans for the measurement without editing scripts. If the beamline is able to overcome simple mistakes such as forgetting to open the shutters before a scan (after a user intervention), the usage of the set-up is more efficient. Automatic alignment options result in reproducible and high-quality data.

In the 1990s the macromolecular crystallography community reacted to the increasing beam time demand with dramatically enhanced automatization and several implementations of method-specific control system frameworks have been described (Gabadinho *et al.*, 2010 ▸; McPhillips *et al.*, 2002 ▸; Bowler *et al.*, 2015 ▸), which maximize usage of the current macromolecular crystallography beamline resources. Most other beamlines, offering methods like diffraction, imaging or XAFS, still lack this extremely high level of optimization.

An implementation of an automatized beamline for this technique is shown on the basis of the XAS beamline at the Karlsruhe Institute of Technology (KIT) synchrotron, which has been in user operation for more than 12 years. This beamline is a bending-magnet beamline (critical energy of 6.23 keV) with only one plane mirror downstream from the double-crystal monochromator. Due to the absence of focusing elements, the short distance from source to sample (14.5 m) and a maximum usable vertical beam size of 1 mm, the set of rules for alignment of the beamline is rather small. To maintain a sophisticated user operation with complex *in situ* experiments handled by a small beamline team, we have constantly upgraded the control system to gain more automation, reliability and automated error detection. The implemented graphical user interface (GUI) was limited to the standard tasks to keep it as simple as possible. This type of reduced interface may restrict a few possible options of the beamline but it gains dramatically in reproducibility and reliability. The control system of the beamline and especially the GUI have to be optimized to the demands of the user community and the capabilities of the hardware. The implementation shown might not be based on the newest open source libraries and sometimes not even on the newest hardware, but this simple implementation is optimized for high throughput of constantly good quality data.

## 2. Implementation   

A significant hurdle is the heterogeneity of the hardware and software environment. Like at many other beamlines, advances in automation were limited mainly by the available time. Therefore the upgrades at the beamline were carried out successively over a long time frame. Nevertheless, only a few limitations in the automation processes are due to the step-by-step implementation of the system. The design and evolution of the control system at the spectroscopy beamlines of the Institute for Photon Science and Synchrotron Radiation (IPS) at KIT were formed based on the respective current requirements. Unlike some other systems, the GUI is implemented as an add-on and never inhibits access to advanced and complex interaction with the beamline.

### 2.1. Hardware automation   

**Figure 1:**
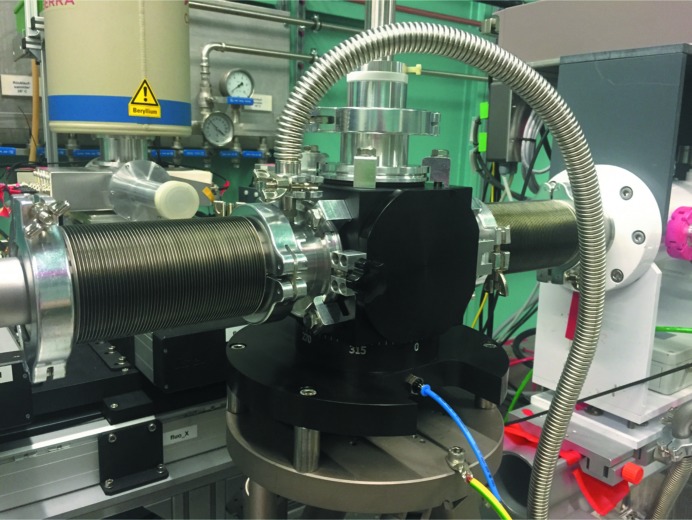
Low-energy vacuum chamber.

One essential requisite of the automation is the required hardware. All standard stepper motors are controlled by OMS MAXv (http://www.omsinmotion.com/maxv) cards, and power supplies of the Middex BCD 130 (http://www.middex.de/motor_schrittmotoren.php?id=3&subid=1) type are used. This set-up is described in more detail elsewhere (Cerff *et al.*, 2014 ▸). For the Bragg axis of the double-crystal monochromator an MCS-8 controller of FMB-Oxford https://www.fmb-oxford.com/products/controls-2/mcs-8-plus-motion-control-system/) is used. The sample positioner for heavy loads (Huber, http://www.xhuber.de/en/) and the Hexapod from PI (https://www.physikinstrumente.com/en/) are operated by dedicated electronics from the respective suppliers. The closed-cycle helium cryostat and the two sample positioners are each installed using two clamping studs of the Zero point clamp system (https://www.zeroclamp.com/en/systems/zero-point-clamping-system/). All other sample equipment is again connected by one Zero Clamp stud to the positioners. Also a vacuum chamber for measurements below 4 keV and measurements with liquid-nitro­gen cooling is easily exchangeable thanks to this system (Fig. 1 ▸).

**Figure 2:**
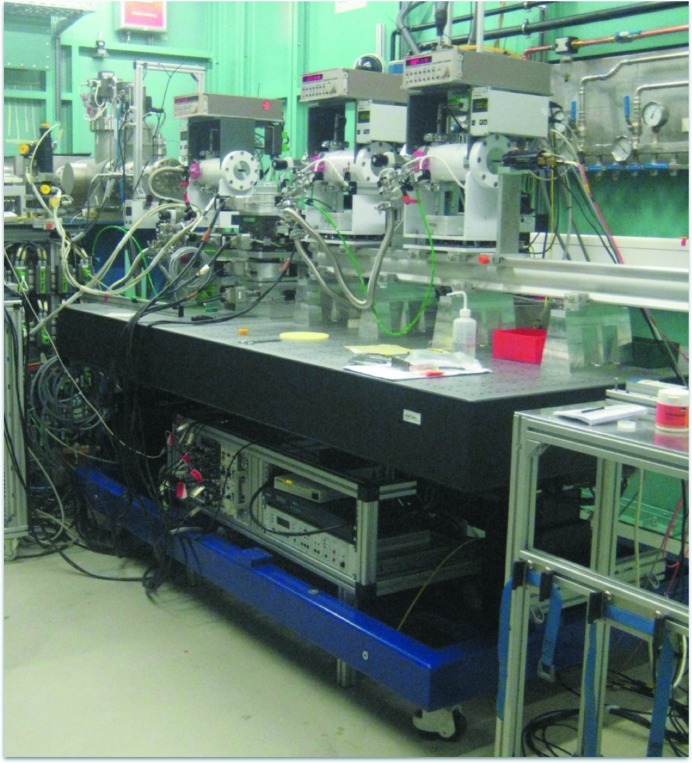
Heavy-load table for standard XAFS usage.

The IC Spec ionization chambers (https://www.fmb-oxford.com/products/detectors-diagnostics/ion-chambers/ic%20spec/) from FMB-Oxford have been specially designed for high-precision X-ray intensity measurements. We use short cables to the Keithley 428 amplifiers (https://de.tek.com/keithley), which are positioned just above the ionization chambers. The voltage is converted by a V2F100 from quantum detectors (http://quantumdetectors.com/v2f100/) to pulses, which are counted by a Struck (http://www.struck.de/sis3820.htm) counter card. The heavy-load experimental table, with six degrees of freedom, was designed and produced in-house at KIT (Fig. 2 ▸). It is optimized for fast positioning of high loads at low backlash, and is able to move rapidly to the pre-defined heights of different setups.

The three fluorescence detectors are exchangeable and adjustable by linear drives. Depending on demand, a one-element silicon drift detector [small size, Vortex (https://www.hitachi-hightech.com/hhs-us/product_detail/?pn=ana-vortex-90ex)], a six-element silicon drift detector with 100 mm^2^ per detector element [Rayspec (https://www.rayspec.co.uk)] and a five-element Ge [Canberra (http://www.canberra.com/products/detectors/germanium-detectors.asp)] are available.

**Figure 3:**
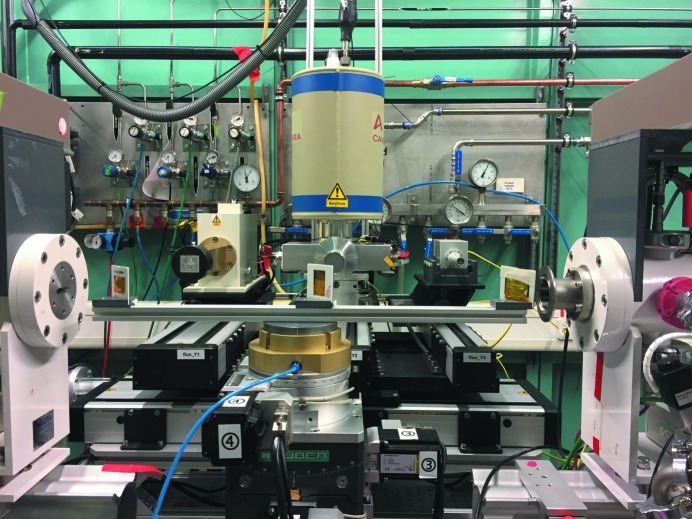
Three fluorescence detectors on linear stages.

The linear drives below the detectors are used for the optimization of the count rate. One linear drive below and perpendicular to these three drives allows the exchange of the detector and the optimization of the detector position along the table. Fig. 3 ▸ shows a metal foil holder for the simultaneous calibration of all three detector systems to each other.

The hardware components of the automation system are listed in Table 1 ▸.

**Figure 4:**
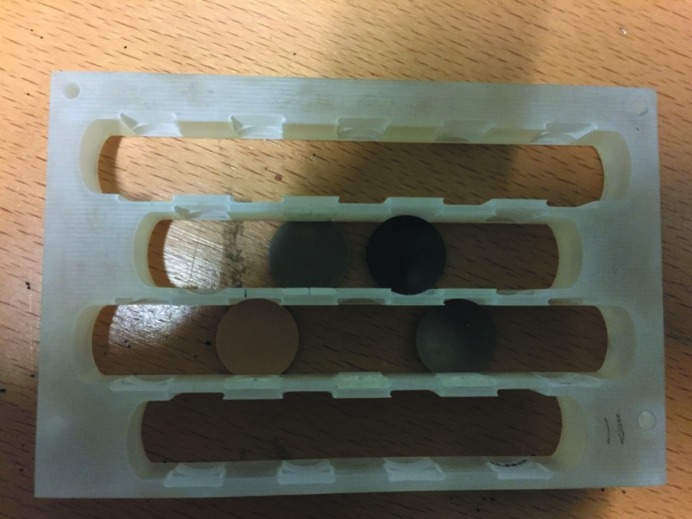
Three-dimensional printed sample holder.

The automatic refilling system of the ionization chambers has operated since 2009/2010 and was implemented during an extended electronic upgrade and hutch extension. During this upgrade the motorized fluorescence detector changer was also installed. Due to time restrictions during this upgrade the heavy-load experiment table and the fast sample-holder/sample-stage exchanger were integrated later. The reproducibility of the sample holders is within 5 µm and allows extremely fast sample exchange and re-measurement of previously measured samples without any new alignment. The three-dimensional printed sample holder (Fig. 4 ▸) has up to 18 pre-defined positions and is used for pellet measurements without any alignment.

### 2.2. Software automation   

#### 2.2.1. Low-level control system software   

The Siemens SIMANTEC S7 system (https://w3.siemens.com/mcms/topics/en/simatic/Pages/default.aspx) is responsible for vacuum and hardware safety while the Pilz-system (https://www.pilz.com/en-GB) handles radiation safety. The Supervisory Control And Data Acquisition (SCADA) system WinCC OA (http://www.etm.at) operates as a slow control for the logging of all systems’ status, vacuum pressures and shutter states. A notification system which acts as a low-level alarm layer is included within this SCADA system. An SMS/E-Mail gateway ensures SMS/E-Mail alarms to the beamline operation manager in case of major hardware issues: typical issues include the liquid-nitro­gen refilling system, the vacuum system and the beamline safety system. The independent data acquisition system is based on *spec* (http://www.certif.com) and Tango components (http://www.tango-controls.org). As a programming platform for users *spec* is quite limited because of the missing name spaces and the drastic consequences of parameter conflicts. For this reason *spec* is only used as a scan- and motor-server (exceptions see Table 2 ▸). For this specific task, however, it still offers unmatched stability and speed compared with a Tango environment (scan- and motor-server). All newer or more complex devices are accessed *via* Tango device servers. The Tango components can be divided into performance non-critical servers like water chillers or high-voltage supplies, servers for motion control (hexapod) and detectors. While the implementation of the first two server types is quite simple, the non-synchronized, parallel and hardware triggered operation of detector servers has to be programmed with great care. Extraordinary measures were taken for the stable operation of the fluorescence detector electronics. During acquisition of continuous XAFS spectra with a duration of 60 s an average of 1250 fluorescence spectra per detector channel are saved. After 23 h this results in more than eight million spectra with the five-element germanium detector. Due to the millions of spectra taken per day in non-patchable libraries, we implemented a special kill-server to restart the XIA libraries in case of issues during server sync after the end of XAFS scans. There is an extremely rare hang-up (once every two to four weeks) at the library level [supplied by XIA (http://www.xia.com/index.html), issues detected also on EPICS-based systems (Christophe Frieh, personal communication)], in combination with some peaking time and silicon drift detectors (*e.g.* 2 µs).

Every piece of software contains several nearly unresolvable errors. These are described in Table 3 ▸.

The simplest way to overcome these kinds of problems is to implement simple countdown timers in several software layers of the control system. Since nearly all of the tasks have a reproducible and well defined duration, the countdown timer can be started with the correct time information. If the execution of a task is blocked for any reason, the timer is not stopped at the successful end of measurement and a SMS/E-Mail alarm is sent out. Two different timer processes (*spec* level and GUI level) are implemented to ensure stable operation of all software levels.

Several provisions are employed to reduce the probability of collisions between beamline components, or broken detector windows. Besides simple hardware and software limits, several other countermeasures are implemented. Simple damage of the detector windows during sample exchange can be avoided by moving the detector out of the measuring position during the closing of the shutters. A set of software limits is implemented taking into account the interplay between different motor movements. These code structures prevent movements of the wrong detectors on the detector changer, help to keep a safe distance between sample environment and detector, and realize a transmission mode with maximum distance to the detectors. If the central detector (Canberra five-element germanium detector; see Fig. 3 ▸) is in the measurement position, none of the two other detectors can be moved towards the middle of the table, and also the movement along the beam direction is limited to ±25 mm. The minimum distance of the fluorescence detector is also dependent on the position of the sample holder.

The sample stages are automatically detected and the non-active device is switched automatically to standby (no motor movement possible). This automatic detection is based on a contact-free resistance measurement of the position of a metal part inside the aluminium baseplate below the sample stages. In addition, several motors are defined as critical motors (high risk of damage) and are normally de-activated at the driver level. By these actions, unintentional movements of motors can be avoided. These motors will be only moved by approved scripts or expert beamline operators.

#### 2.2.2. Basic automation features   

The idea behind any automation at the beamline is to keep things as simple as possible and to divide larger tasks into smaller, reproducible segments. Examples of such scripts are the automatic optimization of gain of the Keithley amplifiers, distance of the fluorescence detectors and correct gas filling of ionization chambers. A number of short procedures continuously detect whether shutters, valves and the storage ring are in a correct state during measurement. Measurement can be started at a specific time or when the storage ring is available after re-injection. A specific naming scheme is used to differentiate between alignment- and measurement-scans. The beamline always writes HDF5 files. The usage of these files by expert users and industry partners was low, therefore we still care about immediate text-file creation. The beamline also offers automatic alignment and refilling of the ionization chambers dependent on the energy. After the optimization of the fixed off-set of the double-crystal monochromator in 2012, the vertical re-alignment is no longer needed for operation from 4 to 20 keV. Since the re-filling of the ionization chambers is the time-limiting step, typically a re-alignment is appended for energy changes of more than 4 keV. One essential reason for the impressive usage of the GUI is the inclusion of beam size and energy changes. These tasks are extremely simple to execute, will stop in case of problems (*e.g.* beam loss of the storage ring) and can be included in complex task sequences. Changes of the vertical beam size keep all three slits optimized with each other. The alignment of both crystals to each other is optimized *via* the second-crystal coarse pitch motor. These optimization steps can be started far away from any optimal beam position and will abort in the event that no increase of the signal strength is achieved (*e.g.* hardware failure).

#### 2.2.3. Top-level control software and GUI   

Two independent applications implemented in *IgorPro* (https://www.wavemetrics.com) are used as a GUI. (*IgorPro* was chosen because of the already available expertise and code.) To ensure a slim user-interface, the functionality is limited to the capabilities of the beamline. Since the code exchange between beamlines at different sources is limited anyway, this choice implies no significant disadvantage. One application handles the input commands; the second is responsible for advanced plotting of the data. Both applications communicate with the beamline *via* two simple and extensively tested TANGO servers. This helps to avoid blocking of data-visualization during extremely large command inputs, and allows the measurement to start immediately after the first input command. To ensure that previously accumulated commands are not immediately executed, rato (the Tango server responsible for the command stack; see Table 2 ▸) does not automatically reconnect to *spec* after unforeseen interruption. Because the GUIs use limited bandwidth they can be used from any computer with network access to the beamline. There is no limit to the number of visualizations, and the command GUI can be launched in parallel, but it requires activation by a sync with the task list server. For safety reasons any out-of-sync communication of the GUIs leads to a halt of the internal core-loops of the *IgorPro* apps. This avoids command-queue corruption and the GUIs are easily restorable by restart and reconnect.

**Figure 5:**
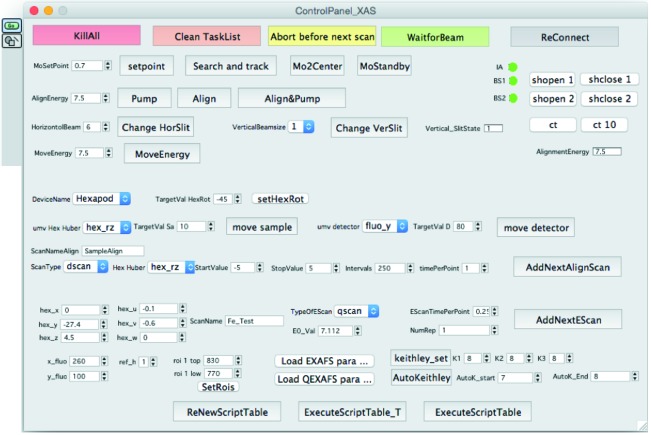
Input panel for the main tasks.

The input GUI (Fig. 5 ▸) maintains an countdown timer for each executed task. The main input GUI checks for the correct and on-time end of a single task to detect any blockage of commands. In contrast to most other beamlines the definition of scans sequences is not based on scripts but on a scheduler-based object-oriented concept. In scripts the change of parameters (detector distance, sample position and reference holder position) is typically inserted on demand, which in our case often resulted in non-optimal detector positions, the incorrect region of interest and other errors. In our implementation the measurement objects have their own parameter set, which is inserted into a table. Each measurement object includes six variables for the position of the sample and one parameter for the reference holder, four settings for the transmission detectors and up to 14 parameters for the fluorescence detectors, two types of scans (continuous and step-scan) and ten different scan definitions per scan-type. This implementation results in a significantly lower number of non-optimal measurements. These types of scan definitions determine several different scan regions around a later specified energy for the scan. Ten scan definitions for each scan type (continuous scan, step-by-step scan) are sufficient for almost any experiment. Typically only the detector distance and the position of the detector in parallel to the beam is changed. Nonetheless, an option for advanced users and beamline staff also allows the change of detector type, minimum allowed detector distance and several different XIA config files to change the peaking times. New tasks can be entered at any time to ensure continuous operation. If there is no demand for additional hardware equipment (*e.g.* vacuum chambers) the beamline can run over extended energy regimes without any re-alignment. Depending on the measurement results the task manager offers the possibility to interrupt or to extend a quick-XAFS measurement sequence.

**Figure 6:**
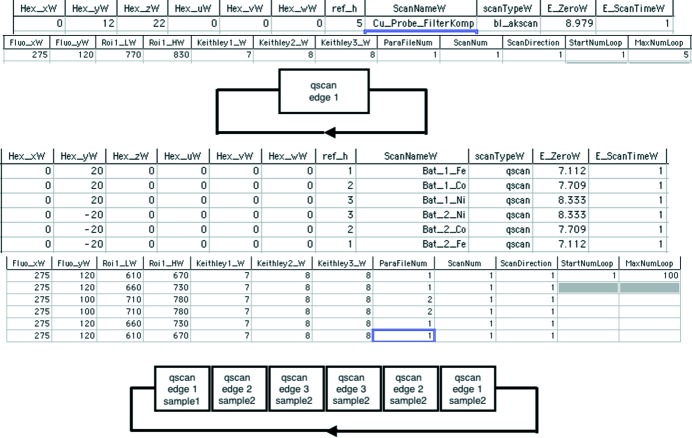
Input table and examples of possible usage.

The input shown in Fig. 6 ▸ allows simple repetitions on one sample, complex sample sets with different repetitions and also a sequence with two batteries with four absorption edges, where for time optimization the edge is measured from low to high energy in one battery and from high to low energy in the other battery.

## 3. Discussion and future extensions   

### 3.1. Conclusions   

The implementation of such a high level of automation requires not only a reasonable investment in time but also a detailed survey of the beamline handling of users. Optimization needs to be done on the basis of the features and usage of the measurement station. Nonetheless, it should be easily possible at any reasonable manpower-equipped third-generation source to implement such an optimized implementation within a time frame of one to two years. The key aim is to transfer simple and boring tasks to the beamline control system, because computers are best for repetitive execution of simple tasks with high reproducibility.

### 3.2. Future possible extensions   

One of the most limiting points is the complexity of measurement stations without having a highly integrated and enforced error detection within the Tango and EPICS control-system layers. Currently most of the externally developed servers have only a limited error detection and even more limited error recovery. The best option would be to have enforced error handling included in the communication handling of the basic control-system components.

To simplify the surveillance of the scheduled tasks, it would be helpful during normal operation to continuously generate movement and settling-times of components to ensure optimized countdown-timers. It would be rather simple to check continuously the flux depending on the beam size or other conditions with an option to send a warning to the beamline staff. In additional to the standard metadata (beamline state, scan type, user ID, *etc.*), the integrity of the data should checked. This could be done by building a Tango bridge [see connection to EPICS (http://pyepics.github.io/pyepics/)] to Larch (Newville, 2013 ▸) and check the reproducibility of several measurements per sample by verifying that the difference between repeated acquisitions is within the noise level. Principle component analysis can be used for this task.

### 3.3. Future extensions for new build-up beamline   

Particularly during the construction of a beamline it would easily be possible to extend the automation even further. During initial build-up it is pretty straightforward to have three-dimensional models of all devices in the sample environment and use these for the collision-protection of beamline and user equipment.

## Figures and Tables

**Table 1:** Hardware automation

Automation type	Needed for	Installation period
Automatic refilling system of the ionization chambers	Automated change of energies	2009/2010 (Mangold *et al.*, 2013 ▸)
Motorized fluorescence detector exchange	Automated optimization of detector distance and exchange of best-suited detectors	2009/2010
Heavy-load experiment table	For handling of the 250 kg detector stage and the fast and automated energy change to low energy	2012–2014
Fast sample holder and sample stages exchange based on Zero Clamp	Measurements of already aligned sample without re-alignment; detection of sample stage for automated standby of unused sample stages	2012–2014
Base one- or two-arm frame of sample holder	Two-arm intermediate holder for heavy sample, one arm holder better for fluorescence	2010
Three-dimensional printed 18-position sample holder	Multi-sample holder with pre-aligned pellet positions	2010

**Table 2:** Tango servers and their tasks

Server name	Main task and usage
ds_Huber_CC	Needed for readout and control of the DCM water cooler
ISEG_NHQ	Needed for the high-voltage supply of the diodes and ionization chambers
ds_TangoLogger	Logging of all user inputs
ds_ANKA_scheme	Needed to change the displayed online data dependent on the actual scan
ds_LocalPublisher	Needed to gain access to the measurement data *via* the Web
ds_PIHexapod	Server for the PI-Hexapod
ds_PIDANKA	Needed for the PID regulation based on National Instruments compactRIO system (Piezo second-crystal DCM)
ds_MCADxpXmap	Needed for loading of configs, Arm for mapping mode, obtaining the raw data in chunks of up to 80 spectra per channel from the digital electronics
ds_xmap_data	Creates all data, which are not produced by the server above during mapping mode (*e.g.* Software-Rois, MCA-Sum)
ds_likeSpecdata	Combines data of point detectors (over counter card), *e.g.* transmission data, with the fluorescence data
rato_Abs	Serves as an interface to obtain data from *spec* and to control *spec* remotely; used for the interface with the visualization based on *IgorPro*
ds_WebCamCapture	Needed for saving webcam images in the data structure
ds_likeSpecHDF5	Generation of HDF5 files
ds_modbusUDP	Modus server
ds_stopCommand	Stops the Xmap server with 100% reliability

**Table 3:** Some nearly unresolvable errors

Type of error	Reason
Rarely occurring memory access error, which depends on race conditions	The performance of the code is typically drastically changed due to the use of the debugger
Rare errors depending on outages of the infrastructure	For control systems of beamlines this is especially difficult because of the dependencies on many external systems
Rare hardware communication issues from controllers, detector electronics *etc*.	The network stack on these components is mostly not state-of-the-art
